# Distribution, Ecological Risk, and Source Identification of Heavy Metal(loid)s in Sediments of a Headwater of Beijiang River Affected by Mining in Southern China

**DOI:** 10.3390/toxics12020117

**Published:** 2024-01-30

**Authors:** Fei Luo, Fawang Zhang, Wenting Zhang, Qibo Huang, Xing Tang

**Affiliations:** 1 Institute of Karst Geology, CAGS/Key Laboratory of Karst Dynamics, MNR&GZAR/International Research Center on Karst under the Auspices of UNESCO, Guilin 541004, China; luofei@mail.cgs.gov.cn (F.L.); huangqibo@mail.cgs.gov.cn (Q.H.); 2 Guangxi Karst Resources and Environment Research Center of Engineering Technology, Guilin 541004, China; 3 Pingguo Guangxi, Karst Ecosystem, National Observation and Research Station, Pingguo 531406, China; 4 Center for Hydrogeology and Environmental Geology Survey, China Geological Survey, Baoding 071051, China; 5 Regional Geological Survey of Guangxi, Guilin 541003, China; wentingzhang11@hotmail.com; 6 Hunan Geological Testing Institute, Changsha 410007, China; xingtanghnt@hotmail.com

**Keywords:** heavy metal(loid)s, sediments, distribution, ecological risk, source analysis, mining activities

## Abstract

In this study, the contents of eight heavy metal(loid)s (As, Pb, Zn, Cd, Cr, Cu, Sb and Tl) in 50 sediment samples from a headwater of Beijiang River were studied to understand their pollution, ecological risk and potential sources. Evaluation indexes including sediment quality guidelines (SDGs), enrichment factor (EF), geo-accumulation index (*I_geo_*), risk assessment code (RAC) and bioavailable metal index (BMI) were used to evaluate the heavy metal(loid)s pollution and ecological risk in the sediments. Pearson’s correlation analysis and principal component analysis were used to identify the sources of heavy metal(loid)s. The results showed that the average concentration of heavy metal(loid)s obviously exceeded the background values, except Cr. Metal(loid)s speciation analysis indicated that Cd, Pb, Cu and Zn were dominated by non-residual fractions, which presented higher bioavailability. The S content in sediments could significantly influence the geochemical fractions of heavy metal(loid)s. As was expected, it had the most adverse biological effect to local aquatic organism, followed by Pb. The EF results demonstrated that As was the most enriched, while Cr showed no enrichment in the sediments. The assessment of *I_geo_* suggested that Cd and As were the most serious threats to the river system, while Cr showed almost no contamination in the sediments. Heavy metal(loid)s in sediments in the mining- and smelting-affected area showed higher bioavailability. According to the results of the above research, the mining activities caused heavier heavy metal(loid)s pollution in the river sediment. Three potential sources of heavy metal(loid)s in sediment were distinguished based on the Pearson’s correlation analysis and PCA, of which Cd, Pb, As, Zn, Sb and Cu were mainly derived from mining activities, Cr was mainly derived from natural sources, Tl was mainly derived from smelting activities.

## 1. Introduction

Heavy metal(loid) contamination in aquatic environments has always been a hot issue in environmental research due to its high toxicity, high persistence, not biodegradability and high bioaccumulation [1], which poses certain harm to the environment [2,3,4]. Heavy metal(loid)s in aquatic environments are mainly released from natural sources (such as soil erosion [5], rock weathering [6]) and anthropogenic activities (such as mining, agriculture, industrialization, transportation, wastewater drainage, fossil fuel combustion and so on [7,8]). Usually, mining activities are considered a major contributor to environmental heavy metal(loid)s [9,10,11]. Once heavy metal(loid)s enter into a river system, they can be rapidly transported for hundreds of kilometers and finally settle in the bottom sediments [2]. When the aquatic environment conditions changed, the heavy metal(loid)s can release into water and cause secondary pollution [7]. Thus, the study of heavy metal(loid)s contamination in river sediment has great significance for ensuring the water safety of a river system [12].

As we know, the total concentration of heavy metal(loid)s in sediments do not effectively reflect their mobility, bioavailability, and toxicity [13]. Numerous studies on heavy metal(loid) contamination in sediments have shown that geochemical fractions significantly affect the mobility, bioavailability, and potential toxicity of heavy metal(loid)s in sediments [14]. The extraction procedure published by the European Community Reference (BCR) classifies heavy metal(loid)s into four fractions (exchangeable, reducible, oxidizable, and residual), which are affected by environmental conditions (e.g., physical-chemical properties of the sediment) [15]. However, most studies have only examined the relationship between total concentration of heavy metal(loid) and environmental conditions or parameters. In general, there are many pollution assessment indexes to evaluate the contamination level and environmental risk [16], such as geo-accumulation index (*I_geo_*) [17], enrichment factor (EF) [18], the risk assessment code (RAC) [19], and especially sediment quality guidelines (SQGs), which are used to characterize the degree of harm to benthic organisms [20]. Thus, different assessment indexes should be used simultaneously to better understand the pollution and environmental risk of heavy metal(loid)s [21]. The origin of heavy metal(loid)s in sediments have often been studied by multivariate statistical techniques, such as PCA and HCA [1,22]. Combining these studies, the distribution and pollution of heavy metal(loid)s could be understood, while the source could be distinguished [23].

The Beijiang River is a tributary of the Pearl River and provides a large amount of water resources for the lower regions, such as mega cities Guangzhou and Shenzhou. In recent decades, the mining and smelting activities rapidly developed in the upper stream, such as in the Fankou Pb-Zn mine, Lechang Pb-Zn mine and Dabaoshan polymetallic mine [24], and the third largest metal producer, the Shaoguan smelter in China [25]. The massive waste tailing slag and wastewater discharged large amounts of heavy metal(loid)s into the river, causing the pollution of the Beijiang River aquatic environment, and it poses a potential risk to the environment and human beings [26]. There was much research about the heavy metal(loid) pollution in sediments in the upstream [27,28] and main river [25], but little focus on the headwater sediments affected by mining activities. In this study, the upper reach of the Wushui River was selected as the study area, which was the headwater of the Beijiang River, and 50 sediment samples were collected from this river. The objectives of this research were (1) to find out the distribution and geochemical fractions characteristics of the heavy metal(loid)s in the surface sediments; (2) to evaluate the contamination and ecological risk level using several indexes; (3) to identify the sources of these metal(loid)s using multivariate statistical analysis. These findings could help to put forward reasonable recommendations and sustainable management for the local river pollution control.

## 2. Materials and Methods

### 2.1. Study Area

Wushui River, as a headwater of the Beijiang river, owns a total length of 270 km and a drainage area of about 7079 km^2^. The study area was in the upper reaches of Wushui river basin, including Linwu County and Yizhang County in southern Hunan Province, which was the key area of the industrial transfer belt in Southern Hunan Province and Northern Guangdong Province, and the river water was usually used as irrigation water. The region belonged to the typical subtropical monsoon humid climate zone, with hot, humid, and rainy summers and dry and less rainy autumns. The landforms in the area were mainly alpine, middle mountain and karst hills. The basin was rich in mineral resources and coal, and its output was mainly tungsten, lead, zinc, tin and other non-ferrous and rare metals. The river basin had many branches, and the mining areas are mainly distributed in the northern basin. Over decades, unreasonable mining activities had led to severe heavy metal(loid) pollution along the Wushui River.

**Figure 1:**
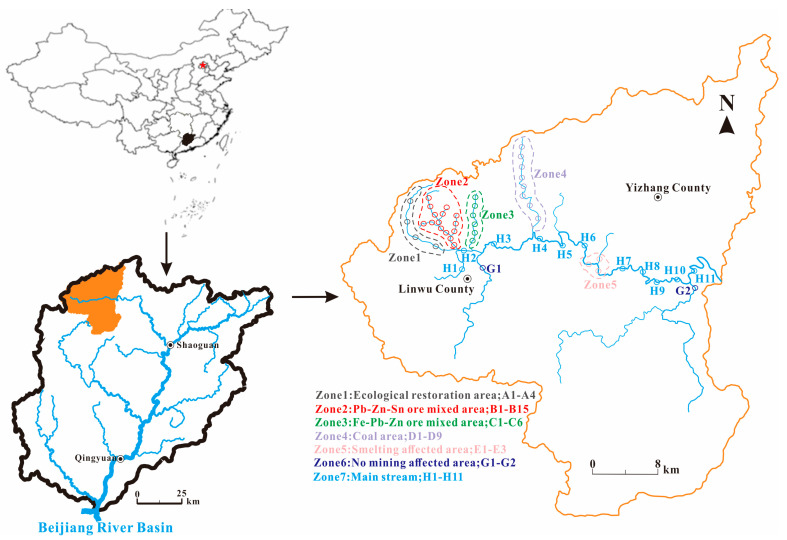
Location of the sampling sites in the Wushui River.

To better understand the impact of mining activities on river sediments, the river basin was divided into the following distinct zones: Zone 1 (ecological restoration area, where there used to be a mining area but now has been restored, the methods of restoration being river channel regulation, building tailings reservoir, and restoring vegetation. The average slope of the branch was 7%, 4 sample sites); Zone 2 (Pb-Zn-Sn ore mixed area, where the branches take on the shape of a tree and own a lot of Pb-Zn ore, Sn ore, including mining, processing, and disposal zones from the upstream to downstream. The average slope of the branch was 6.5%, 15 sample sites); Zone 3 (Fe, Pb-Zn ore mixed area, where the mining operations concentrated in the upstream of the branch and owned an average slope of 8.5%, 6 sample sites); Zone 4 (coal area, where the mining operations were concentrated in the upstream of the branch and owned an average slope of 12.5%, 9 sample sites); Zone 5 (smelting affected area, which is mainly affected by metal smelting, 3 sample sites); Zone 6 (no mining affected area, two tributaries with little human activity, 2 sample sites); Zone 7 (the main stream of the Wushui river, 11 sample sites) (Figure 1).

### 2.2. Sediment Sampling and Analytical Procedures

#### 2.2.1. Sample Collection and Preparation

Fifty samples were collected in the Wushui River from the seven different zones during July 2020. Sampling sites are showed in Figure 1. The sediments with a depth of 0~5 cm were collected by a Van Veen grab sampler (ETC200). At each site, 3 sub-samples were collected and homogenized to obtain a composite sample with a weight of 1 kg. The sampling sites were positioned by a global positioning system (GPS). After collection, the sediment samples were put into the polyethylene plastic bags and stored in an insulated box with ice packs. The samples were then transported to the laboratory quickly and air-dried to remove impurities such as gravels, plant, plastic. An agate mortar and pestle were used to grind the dried samples, then filtered through a 200-mesh nylon sieve and prepared for measurement [29].

#### 2.2.2. Analytical Procedures

The sediment pH was measured using a mixture of 15 mL deionized water and 6 g sediment stirred with a glass rod for half an hour, then measured by the IS128C pH meter (Insmark Ltd., Shanghai, China). The granular matrix index of sediment was determined by the settlement method [4], and finally, the sand/silt/clay ratio was calculated. Total organic carbon (TOC) content was determined by a TOC analyzer. S content was determined using a Perkin Elmer 2400 CHNS/O analyzer (Norwalk, CT). Major element content of the sediment samples was measured using XRF (ZSX Primus II), wherein absolute errors of Si and Al were ±0.5% and ±0.2%, while the errors of other elements were below 10%.

To extract the total content of heavy metal(loid)s, the pre-treated sediment samples were digested by a mixture of acids consisting of HNO_3_-HCl-HF-HClO_4_ solution in a microwave [30]; then, the concentrations of eight heavy metal(loid)s (As, Pb, Zn, Cd, Cr, Cu, Sb and Tl) in the extract were determined via ICP-MS (inductively coupled plasma mass spectroscopy, Agilent 7500 Series).

Sequential extraction was an effective method to elevate the mobility scale of the metals in the soil and sediments. In this study, a modified BCR sequential extraction procedure was used to divide metals into four fractions defined as the acid-soluble/exchangeable fraction (F1), reducible fraction (F2), oxidizable fraction (F3) and residual fraction (F4) [31]. After each extraction process, the supernatant was filtrated through a 0.45-μm fiber membrane before determining the available heavy metal(loid)s using ICP-MS.

#### 2.2.3. Quality Assurance and Quality Control

Samples were conducted in strict accordance with quality assurance/quality control (QA/QC) measures. To ensure quality accuracy, these samples were analyzed in duplicate with a relative standard error (RSD) of ±5%. For better control of the quality of the analysis, the standard soil (GBW07425) was added, and the recoveries for metal(loid) contents were between 90 and 105%. The analytical detection limits of As, Pb, Zn, Cd, Cr, Cu, Sb and Tl in the current study were 0.0003 mg kg^−1^, 0.0006 mg kg^−1^, 0.022 mg kg^−1^, 0.003 mg kg^−1^, 0.022 mg kg^−1^, 0.055 mg kg^−1^, 0.0004 mg kg^−1^ and 0.0008 mg kg^−1^, respectively. All the analyses were conducted in the Hunan Geological Testing Institute.

### 2.3. Data Analysis and Risk Assessment

#### 2.3.1. Statical Analysis

The sampling location map was made by CorelDraw 2018. The data were analyzed by SPSS 21.0 and EXCEL and plotted by Origin 2022. Before conducting a statistical analysis of the data, Shapiro–Wilk was applied to examine the data normality and homoscedasticity. The relationship among sediment properties, heavy metal(loid)s concentration and fraction were evaluated using the Pearson correlation matrix (PCM) method.

#### 2.3.2. Sediment Quality Guidelines

In this study, the probable effect concentrations (PEC) and the threshold effect concentrations (TEC) were used because there were no sediment quality regulations in the study area [32]. PEC indicated the concentration above which adverse effects were expected to frequently occur, while TEC meant the concentration below which adverse effects rarely occur [33].

#### 2.3.3. Geo-Accumulation Index

The geo-accumulation index (*I_geo_*) was used to quantify metal contamination caused by natural geological processes and human activities [34], and was calculated by the following formula:

*I_geo_* = log_2_[*C_m_*/(1.5*B_m_*)] (1)

where *C_m_* is the concentration of metals of the target samples, and *B_m_* is the background level of the evaluated metal in the study area. The adjustment coefficient for lithospheric effects is 1.5. The detailed heavy metal(loid) contamination level is listed in Table S1.

#### 2.3.4. Enrichment Factor

The enrichment factor (EF) was usually used to calculate the degree of anthropogenic heavy metal(loid)s pollution [35], and was calculated according to the following equation:

*EF* = [*(C_E_*/*C_R_*) *_Sample_*]/[(*C_E_*/*C_R_*) *_Background_*] (2)

where (*C_E_/C_R_*) *_Sample_* represents the ratio between the level of the examined metal and the level of a reference element in the river sediment, and (*C_E_/C_R_*) *_Background_* is the ratio of two elements in the study area background soil. In this study, we chose the Al_2_O_3_ as reference element because Al is chemically stable, and its concentration shows little difference at different sites. The detail heavy metal(loid) contamination level listed in Table S1.

#### 2.3.5. The Risk Assessment Code

The Risk Assessment Code (RAC) was used to assess the ecological risk of each metal, which was defined as the ratio of the acid-soluble fraction (BRC-F1) in the heavy metal(loid) total content [36,37]. RAC was calculated by the Equation (3):

*RAC* = *C_F*1*_*/*C_i_* × 100% (3)

where *C_F*1*_* (mg/kg) represented the heavy metal(loid) *i* content in F1 fraction, and *Ci* (mg/kg) represented the total content of heavy metal(loid) *i*. The detail classification of RAC was listed in Table S1.

#### 2.3.6. Bioavailable Metal Index

The bioavailable metal index (BMI) was usually used to assess the bioavailability of the heavy metal(loid)s in the sediments [38]. BMI was calculated by Equation (4):

*BMI* = [*C_F*1*_*^1^/*C*^1^*_R_* × … × *C_F1_*^i^/*C*^i^*_R_* × … × *C_F1_*^n^/*C*^n^*_R_* ]^(1/n)^ (4)

where *C_F*1*_*^i^ represented the bioavailable concentration (BCR-F1) of heavy metal(loid) I in sample, *C*^i^*_R_* represented the bioavailable concentration of heavy metal(loid) I in the reference background sample, and n was the number of heavy metal(loid)s investigated. Site G2 was chosen as the background sample because the heavy metal(loid)s at this site were lower than in other sampling sites and were close to the background values.

## 3. Results and Discussion

### 3.1. Sediment Properties and the Distribution of Heavy Metal(loid)s

#### 3.1.1. Sediment Physical–Chemical Properties

The sediment physical–chemical properties (e.g., pH, TOC, TN, S and particle size) had a significant impact on the release, mobility, and availability of heavy metal(loid)s in river sediments [4,39]. As shown in Table S2, the river sediments had a pH value of 3.1~10.6 and an average pH value of 7.7, which was weak alkaline and reflected the typical characteristics of a river in karst area [40]. The pH value of the sediment in the study area was lower near the mine site and higher downstream. The S content ranged from 0.03% to 4.50%, with a mean value of 0.48%. The highest S content was found in Zone 3, and there was a trend of higher S content in mining-affected areas. The total nitrogen (TN) content in the sediment varied with the sites and ranged from 117 to 3860 mg kg^−1^, which was lower than the values in the Wuxi river sediments in Taihu Lake in China [41] and the Thamirabharani Indian river [4] because the study area was the headwater of the river basin and had less agriculture runoff and sewage effluent [42,43]. The TN in the sediment showed the trend that the upstream affected by mining had the lower value, and the mainstream (Zone 7) had the highest average TN value. The TOC ranged from 0.24% to 2.87% and with an average value 1.33%, which was lower than that of Huaihe River [44] and higher than that of Thamirabharani River [4] and Sava River [39]. The highest TOC value was found in Zone 4 because of coal mining activities, while the other areas had lower TOC values, especially in the mining and smelting areas. The average composition content of the sediments was sand at 77.13%, silt at 20.32% and clay at 8.56%. Compared to other studies [4], the relatively low clay content in the sediments did not show a common trend of fluctuating from upstream to downstream sediments with decreasing sand content and increasing silt and clay content. For example, sediments in Zone 2 and Zone 3 contained higher content of silt and clay than total basin, especially Zone 3, which contained the highest content of silt and clay. This may be due to increased soil erosion caused by mining, which deposited finer particles.

#### 3.1.2. Concentrations and Spatial Distributions of Heavy Metal(loid)s in Sediments

As showed in Table 1, the mean concentrations of heavy metal(loid)s varied widely and dramatically among the sampling sites and ranked as As > Zn > Pb > Cu > Cr > Sb > Cd > Tl. Compared with the background concentration of sediment heavy metal(loid)s in Pearl River [45], the average concentration of heavy metal(loid)s obviously exceeded their background values, except for Cr. The average concentrations of Cu and Tl were slightly over their background values by 2.31 and 2.98 times, while Cd, As, Sb, Pb, Zn were significantly over their background values by 75.67, 68.02, 25.34, 20.44, 10.21 times, which indicated that the aquatic environment of the Wushui River was seriously polluted by heavy metal(loid)s, especially Cd and As, which is extremely serious. In addition, the content of heavy metal(loid)s in sediment showed a huge concentration variation, and the coefficient of variation (CV) ranked as Sb (1.36) > Pb (1.33) > As (1.07) > Cd (1.05) > Zn (0.99) > Cu (0.78) > Tl (0.73) > Cr (0.34), which indicated that the heavy metal(loid) content varied spatially within the study area. An element showed a high CV value maybe due to the presence of point source pollution or a large variation in its sources within the region [9], which was consistent with the fact that there were many mining activities in the study area.

**Figure 2:**
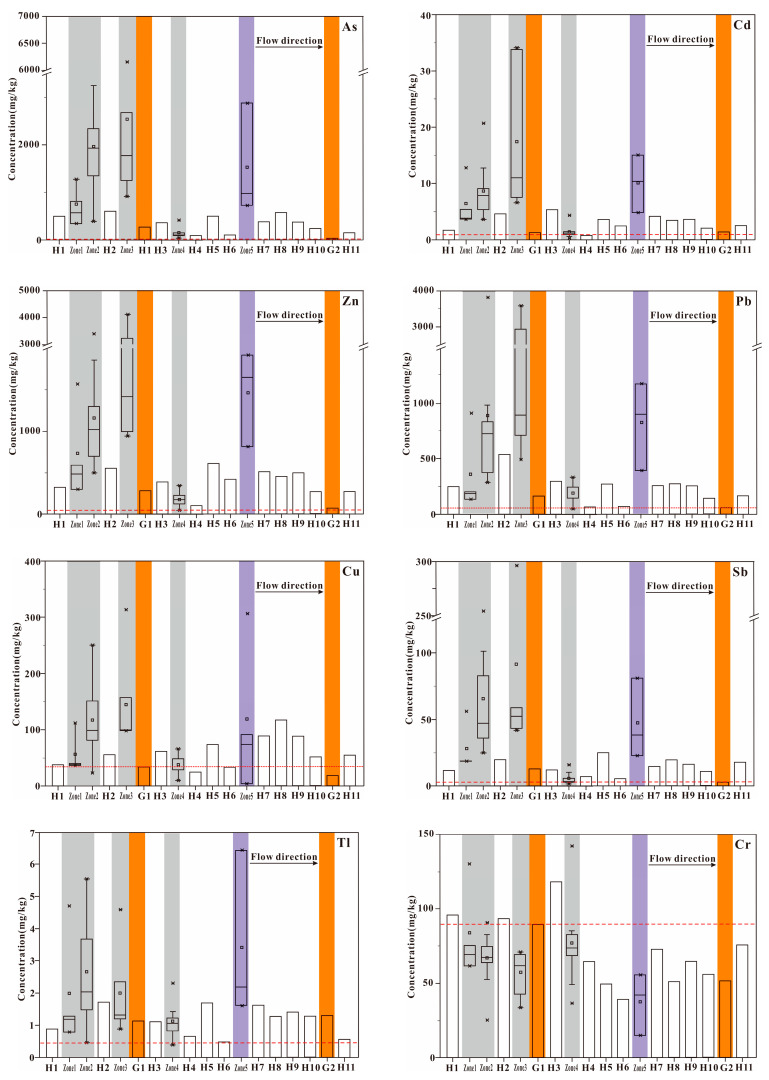
The distribution of heavy metal(loid)s in sediments of Wushui River. The three horizontal lines from lower to upper in the box represent quartile of 25% 50%, and 75%, respectively, square dot represents the mean value, and star point represents outside whisker. The red dotted line represents the background values of heavy metal(loid)s. The gray area represents the sampling sites in the mining affected area, the purple area represents the sampling sites in the smelting affected area, the orange area represents the sampling sites in the no mining affected area.

For a better understanding of the distribution of heavy metal(loid)s in the Wushui River, the study area was divided into seven zones according to their location and type of pollution source. According to Table 1, the tributaries with mines exhibited an extremely high content of heavy metal(loid)s in the sediments. Zone 2 and Zone 3 were heavily impacted by mining activities with high As, Cd, Zn, Pb, Sb in the riverbed sediments; Zone 5 was the smelting area that also showed high heavy metal(loid) content and the highest Tl in sediments. After ecological restoration, heavy metal(loid)s in Zone 1 were significantly decreased (50~70% compared to Zone 2 and Zone 3), which showed a remarkable environmental change. In the coal mining impacted area (Zone 4), due to the large slope of the stream and low concentration of heavy metal(loid)s in coal, there was a lower concentration of heavy metal(loid)s in the sediments. The two tributaries with no mining activities (G1 and G2) exhibited relatively low As, Cd, Zn, Pb and Sb in the sediments, whereas more anthropogenic activities upstream of G1 had resulted in higher levels of heavy metal(loid)s in the sediments. The mean content of heavy metal(loid)s in the mainstream sediments were significantly lower than that in the areas affected by mining activities and the smelting area, and the content showed a decreasing trend with the downstream distance [40], but the content of heavy metal(loid)s increased slightly after the tributaries affected by mining merged into the mainstream (Figure 2).

Compared with other watersheds in the Pearl River Basin (Table 2), the river with mining activities exhibited a higher As and Pb concentration in this study than other mining-impacted rivers, such as the Diaojiao River [40] and Hengshi River [28], while these rivers were severe polluted by mining. The content of heavy metal(loid)s, except Cr in the sediments of mainstream of Wushui River, were obviously higher than in the mainstream of Pearl River and the background values of Pearl River; this evidence suggested that the Wushui River basin had been seriously polluted due to mining.

#### 3.1.3. Chemical Fractions of Heavy Metal(loid)s

**Figure 3:**
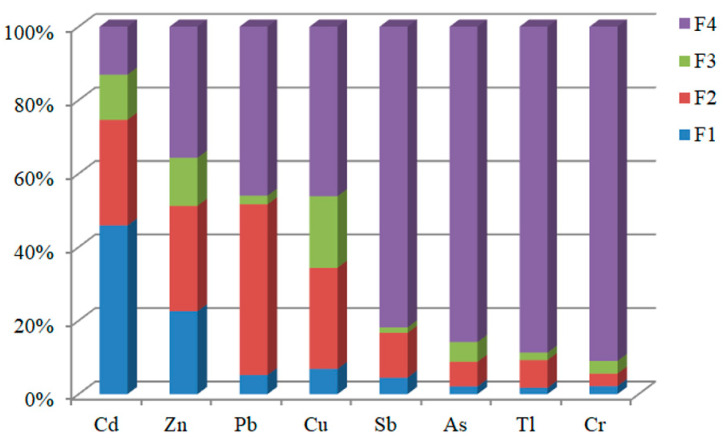
BCR speciation of heavy metal(loid)s (mean value) in surface sediments of Wushui River.

The chemical fractions of heavy metal(loid)s in the sediments significantly affected their mobility, bioavailability and biotoxicity [46,47]. The BCR extraction fraction (F1, F2, F3 and F4) results showed that the geochemical fractions of heavy metal(loid)s varied with species and sites (Figure 3). The heavy metal(loid)s could be divided into four categories according to the main fractions: Cd dominated the F1 fractions (45.91%, 14.25~83.57%), Pb dominated the F2 fractions (46.44%, 10.61~72.95%), Cu and Zn dominated the F2 + F3 fractions (47.01% and 41.77%, 9.26~85.26% and 10.05~80.26%), while the other metals (Sb, As, Tl, Cr) dominated the F4 fractions (81.82%, 85.82%, 88.68%, 90.96%). Firstly, Cd had relatively higher F1 fractions than other metals, which indicated Cd was easily displaced by cations (e.g., K^+^, Ca^2+^, Mg^2+^) and released [48], and became more bioavailable [38] and posed direct harm to aquatic organisms. Pb dominated the F2, which was the reducible fraction of metal bound to Fe and Mn oxy/hydroxides, which could release to the water environment when the aquatic system goes into the reducing condition [18]. Cu and Zn dominated the F2 + F3, which were bound to Fe-Mn oxides, organic matter, and sulphides. When the redox conditions of the aquatic system changed, Cu and Zn could release into the water and pose a serious threat to the water environment [49]. The distribution for the chemical fractions of Sb, As, Tl, and Cr in the sediments were dominated in the residual fraction (F4) bound to aluminosilicate minerals, which indicated these metal(loid)s showed low mobility and bioavailability [50], thus presenting little harm to the aquatic environment. Although As mainly existed in the F4 fraction, its bioavailable content (F1 + F2 + F3) was high, which also owned certain environmental risk.

The distribution fractions of heavy metal(loid)s in the sediments changed spatially (Figure S1). Except for site H 2, H 6, and H 11, where cadmium was mainly of the F2 fraction, cadmium at the other sampling sites was mainly of the F1 fraction and showed a trend wherein after the tributaries in the mining-affected area merged into the main river, the F1 fraction increased slightly, while downstream it gradually decreased due to the alkaline nature of the river water in the karst areas. The distribution of Zn and Cu had a similar trend mainly in the F4 fractions in the slightly human activity-affected tributaries.

#### 3.1.4. Factors Influencing the Fractions of Heavy Metal(loid)s

The physical–chemical properties of sediment could affect the geochemical fractions of heavy metal(loid)s [41]. Thus, the PCM (Pearson correlation matrix) method was used to systematically analyze the correlations between fractions and sediment properties for the selected six heavy metal(loid)s (As, Cd, Pb, Zn, Sb and Cu), which were heavier polluted (Table 3).

The lower pH could promote the mobilization of heavy metal(loid)s-the F1 fraction, which bound to carbonates and changed the geochemical fraction in the sediments. According to Table S1, the mean pH of the study sediments was 7.70, indicating that the sediments from Wushui River were almost alkaline. Thus, the pH of the sediments had little effect on the fractions of heavy metal(loid)s, and there was a weak correlation between the pH and the fraction of heavy metal(loid)s [38]. The particle size of the sediment could affect the distribution and fractions of heavy metal(loid)s in the sediments [51]. As shown in Table 3, silt and clay showed a significant positive correlation with the F1 and F2 fractions of Cu, while sand showed a negative correlation with the F1 and F2 fractions of Cu, suggesting that small sediment particles could increase copper mobility and bioavailability [30]. Other heavy metal(loid)s showed weak correlations with the particle size of sediments. TOC was often regarded as the main carrier of heavy metal(loid)s and could affect the migration and transformation of heavy metal(loid)s due to the complexation of TOC with heavy metal(loid)s [52]. According to Table 3, the weak correlations between the TOC and the heavy metal(loid) fractions indicated that TOC was not the main factor influencing the heavy metal(loid) fractions, which may be due to the low TOC content (average value 1.33 mg/kg, ranged from 0.24 mg/kg to 2.87 mg/kg) in the sediment. The negative correlations between TN and the fractions of heavy metal(loid)s implied that they may have the same sources, mutual dependence, and transport behavior [14]. Additionally, S played a crucial role in controlling the fractions and distribution of the selected six heavy metal(loid)s; there were significant positive correlation between Cu-F4, Pb-F4, Zn-F4, Cd-F4, As-F4 and Sb-F4 in the sediments, which implied that the S content could significantly reduce the mobility and bioavailability of heavy metal(loid)s in sediments. According to previous studies, S content always showed significantly positive correlations with F3 fractions because the F3 fraction was bound to organic matter and sulphide; thus, S and heavy metal(loid)s may form stable complexes that belong to residual fractions in the sediments [53].

### 3.2. Pollution and Risk Assessment for Heavy Metal(loid)s

#### 3.2.1. SQGs

The biological effects of heavy metal(loid)s in sediment could be assessed by comparing those concentrations with SQGs. The compared result between TECs and PECs and heavy metal(loid)s in sediments were shown in Table 4. Tl and Sb had no data due to the lack of the TEC and PEC date. Cr and Cu had similar distribution patterns, which, mainly between TECs and PECs, indicated that Cr and Cu pose potential biological effects to local aquatic organisms. The other four metal(loid)s’ concentrations were almost half of the sample sites and exceeded the PECs, especially since As in 49 sites (98% of samples) and Pb in 41 sites (82% of samples) exceeded the PECs, which were expected to have adverse biological effect on local aquatic organisms. In addition, the background values of the heavy metal(loid)s were lower than that of the TECs, which suggested that the sediments in the study area may be enriched with heavy metal(loid)s.

#### 3.2.2. Enrichment Factor

The results of the EF index values for the Wushui river are shown in Table 5. The average EF value of the selected heavy metal(loid)s was As > Cd > Sb > Pb > Zn > Cu > Tl > Cr. The results show that cadmium and arsenic were generally more enriched than other heavy metal(loid)s. The EF value of Cd ranged from 3.98 to 801.4, with an average value of 78.09, and 48 sample sites were beyond 5, indicating significant enrichment of Cd in these sediments. The EF value of As ranged from 2.43 to 1135.63, with an average value 96.76, and 45 sample sites were beyond 5, also indicating significant enrichment in the sediments. The average EF values of Sb and Pb were 32.35 and 28.82, indicating that the percentage of significant enrichment for these two metals was low, and the distribution trends were similar for each level of contamination. The average EF values of Zn, Cu and Tl were 11.29, 3.69 and 2.74, which presented the significant enrichment and moderate enrichment. The EF value of Cr ranged from 0.13 to 1.68, with an average value 0.81, and indicated deficient enrichment and moderate enrichment in the sediment, while most sites were deficient enrichment. The analysis of the spatial distribution of the EF values showed that mining-affected areas were more heavily contaminated with heavy metals, which indicated that the pollutants mainly come from mining activities.

#### 3.2.3. Assessment by *I_geo_* Index

The results of the *I_geo_* value of heavy metal(loid)s are shown in Table 5. Obviously, Cd and As were heavy polluters, with average *I_geo_* values of 4.23 and 4.07, and about 90% of the samples sites had a *I_geo_* value > 2.0, especially in the mining-affected area (zone 2, zone 3), which were extremely polluted. Cr showed no contamination in the river sediments (*I_geo_* < 0), except in two sites (0.06, 0.19) in the mining-affected area. Tl and Cu had similar *I_geo_* values, with approximately half of the samples having a value between 1.0 and 3.0 in Zone 2 and Zone 3, while the other sites had lower *I_geo_* values, implying slight pollution. Pb, Zn and Sb were mostly biased towards moderate contamination (2.0 < *I_geo_* < 3.0) in the main river and Zone 1, and had moderate or heavy contamination in the mining area, except in Zone 4. These results indicate that the main polluted heavy metal(loid)s in the sediments of Wushui river came from Cd and As, which was consistent with the research results from Xiangjiang river [54] and the Pearl River [23]. Based on the data, we found that the mining-affected areas had higher *I_geo_* values, except for Cr, when these tributaries joined the main river, which caused a promotion, and then declined gradually; such spatial variations in heavy metal(loid)s contamination in sediment were significantly useful for environmental protection and for predicting heavy metal(loid) patterns in the watershed.

#### 3.2.4. Assessment by RAC

The RAC value based on metal BCR-F1 friction was used for evaluating the bioavailability and migration of heavy metal(loid)s in the sediments [55]. As showed in Table 5, the average RAC values are listed in the following order: Cd > Zn > Cu > Pb > Sb > Cr > Tl > As, showing that Cd and Zn were the main potential ecological risks to the river system. Other heavy metal(loid)s exhibited slight ecological risk. The RAC values of Cd ranged from 14.25% to 83.57%, while 38% of the sites showed an RAC value >50%, and 56% of the sites showed an RAC value between 30% and 50%, which demonstrated that Cd poses the heaviest ecological risk. The RAC values of Zn ranged from 3.89% to 62.99%, while 4% of the sites showed an RAC value > 50%, and 22% of the sites showed an RAC value between 30% and 50%, which indicated that Zn could also cause some ecological risks. Most of the sample sites in the mining-affected area presented higher RAC values. Although the other four metals showed slight ecological risks according to the RAC value, we should pay attention to As since the F1 fraction of As clearly exceeded the background value in the study area.

#### 3.2.5. Assessment by BMI

Figure S2 showed the BMI values for each sampling site (the BMI value of Zone 1–Zone 5 was the mean value of the sampling sites in each area). According to Figure S2, the bioavailability of the sampling sites was higher in the mining-affected area and smelting-affected area. With the direction of the river, the BMI value decreased gradually, and when there was a tributary in the mining-affected area or a tributary in the smelting-affected area that was converging, the BMI increased to a certain extent, and then decreased gradually. Previous studies supposed that the bioavailable fraction of different heavy metal(loid)s was positively related to their toxic effects [38]. Thus, the high bioavailability of heavy metal(loid)s in the mining- and smelting-affected area represented high sediment toxicity, which had a serious impact on the benthic organisms.

### 3.3. Sources Analysis of Heavy Metal(loid)s

**Figure 4:**
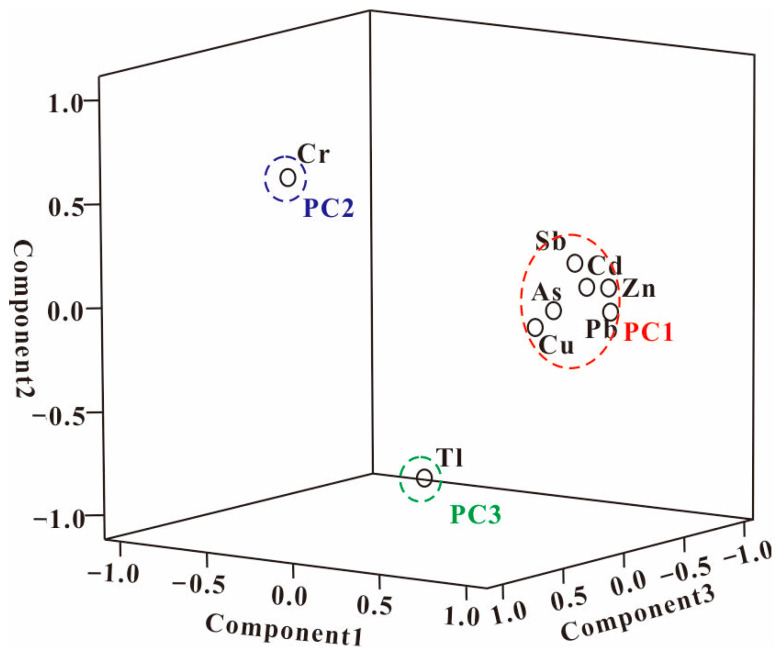
Principal components analysis loadings and score plots for the heavy metal(loid)s.

To identify the relationship and the potential sources of heavy metal(loid)s in the sediments of Wushui River, Pearson’s correlation coefficient analysis and PCA were applied [56]. The good correlations between the heavy metal(loid)s in the sediments could reveal similar sources. The Pearson’s correlation matrix was conducted, and the correlation coefficients are shown in Table S3. Cu, Zn, Pb, As, Sb and Cd were obviously positively correlated with each other, showing that these heavy metal(loid)s could have originated from the same source. Tl and Cr showed no positive correlation with each other and other heavy metal(loid)s, suggesting that these two metals came from different sources. Further identification of the sources of measured heavy metal(loid)s was used by the PCA approach. PCA was an effective method for the source analysis of the heavy metal(loid)s [56]. The results of Keiser–Meyer–Olkin (0.691) and Bartlett’s tests (<0.001) indicated that the PCA analysis was valid. As showed in Table S4, 86.72% of the cumulative variance could be explained by three principal components (PCs) (Figure 4). PC1 accounts for 61.462% of the total variance and could be explained by the high loading of Cd (r = 0.913), Pb (r = 0.907), As (r = 0.892), Zn (r = 0.889), Sb (r = 0.874) and Cu (r = 0.806). According to the correlation coefficients, these six metals(loid)s had the same sources, and these metal(loid)s were heavily affected by the mining activities; thus, PC1 could possibly be due to the mining activities of the mining area. Previous research has indicated that mining activities could produce large quantities of contaminants composed of various waste rocks, slag, fine-grained ore minerals, ore weathering products such as stibnite, and As-alkaline residues [57]. All these wastes were discharged into the surrounding environment and finally deposited into the river sediment, causing heavy metal(loid) pollution. PC2 explained 13.440% of the total variance and showed high loading for Cr (r = 0.627) only. The low concentrations and low CVs in the sediments (close to the background values) suggested that Cr mainly originated from rock weathering and atmospheric precipitation, which was similar to previous studies [38]. PC3 was dominated by Tl (r = 0.600), with 10.306% of the total variance. The high concentrations and high *I_geo_* values were mostly in Zone 5 (smelting affect area), indicating that Tl mainly formed the smelting. According to a previous study, Sb and its oxides were emitted as submicron particles and gases into the atmosphere during the smelting processes and could be transported into river sediment [27].

## 4. Conclusions

The research on As, Zn, Pb, Cu, Cr, Sb, Cd, and Tl in the sediments from the Wushui River suggested that As concentration was the highest and Tl concentration was the lowest. The average concentration of heavy metal(loid)s obviously exceeded the background values, except for that of Cr. The BCR extraction results showed that Cd mainly dominates in the F1 fractions, Pb in the F2 fractions, and Cd and Zn in the F2 + F3 fractions, while the other metal(loid)s dominate in the F4 fractions. The S content in sediments could significantly influence the geochemical fractions of heavy metal(loid)s. Comparing heavy metal(loid) concentration with SQGs, As was expected to have the most adverse biological effect on the local aquatic organisms, followed by Pb. According to the EF results, the heavy metal contamination was ordered As > Cd > Sb > Pb > Zn > Cu > Tl > Cr, and Cr showed no enrichment in the sediments. The assessment of *I_geo_* suggested that Cd and As were the most serious threat to the river system, while Cr showed almost no contamination in the sediments. The results of the RAC showed that the potential mobility and bioavailability of Cd and Zn were much higher than those of other heavy metal(loid)s. The heavy metal(loid)s of the sediments in the mining- and smelting-affected area showed higher bioavailability. Synthesizing the results of the above research, the mining activities caused heavier heavy metal(loid) pollution in the river sediment. The potential sources were identified by Pearson’s correlation analysis and PCA, combed with the pollution characteristics; Cd, Pb, As, Zn, Sb and Cu were mainly derived from mining activities; Cr was mainly derived from natural sources; and Tl was mainly derived from smelting activities. This study effectively grasped the status quo of heavy metal(loid) pollution in the Wushui River sediment under the influence of mining activities and could put forward reasonable ecological protection measures.

## Figures and Tables

**Table 1:** Total heavy metal(loid) concentration ranges in the sediments from Wushui river.

River	Heavy Metal(loid) Concentration (mg/kg)								
	Item	Cu	Pb	Zn	Cr	Cd	As	Sb	Tl
Zone 1 (n = 4)	Minimum	36.00	136.00	299.00	61.70	3.70	345.00	18.40	0.78
	Maximum	112.00	916.00	1570.00	130.00	12.80	1270.00	55.80	4.70
	Average	56.48	359.75	736.25	84.08	6.45	745.50	27.93	1.98
	CV (%)	65.61	103.37	77.27	37.01	66.73	53.17	66.55	92.01
Zone 2 (n = 15)	Minimum	22.90	287.00	498.00	25.40	3.62	389.00	24.90	0.45
	Maximum	250.00	3810.00	3410.00	90.80	20.70	4400.00	254.00	5.54
	Average	117.13	892.53	1158.73	67.01	8.65	1962.60	65.25	2.65
	CV (%)	55.48	99.95	62.00	22.02	53.63	50.13	87.72	55.02
Zone 3 (n = 6)	Minimum	98.20	495.00	943.00	33.70	6.63	911.00	41.80	0.88
	Maximum	313.00	3580.00	4120.00	70.90	34.10	6150.00	296.00	4.59
	Average	144.02	1618.33	2030.00	57.43	17.48	2538.50	91.35	1.99
	CV (%)	59.73	80.28	65.27	26.84	73.88	74.70	110.00	68.84
Zone 4 (n = 9)	Minimum	9.50	21.40	42.70	36.60	0.31	32.10	1.36	0.38
	Maximum	65.70	220.00	345.00	142.00	4.37	413.00	15.80	2.30
	Average	37.43	112.94	176.17	76.80	1.46	150.74	5.33	1.11
	CV (%)	45.42	69.16	53.43	38.14	80.71	78.49	87.98	47.89
Zone 5 (n = 3)	Minimum	73.70	397.00	819.00	15.00	4.82	722.00	22.80	1.60
	Maximum	306.00	1180.00	1920.00	55.60	15.10	2880.00	80.90	6.43
	Average	157.13	828.33	1463.00	37.57	10.11	1526.00	47.30	3.40
	CV (%)	82.25	47.99	39.22	55.04	50.92	77.29	63.64	77.49
Zone 6 (n = 2)	Minimum	18.60	55.20	113.00	51.70	1.22	38.60	2.54	1.14
	Maximum	33.50	162.00	306.00	89.50	1.35	270.00	12.70	1.31
	Average	26.05	108.60	209.50	70.60	1.29	154.30	7.62	1.23
	CV (%)	40.44	69.54	65.14	37.86	7.15	106.04	94.28	9.81
Zone 7 (n = 11)	Minimum	24.70	61.40	139.00	39.30	0.74	96.40	5.16	0.48
	Maximum	117.00	370.00	600.00	118.00	5.31	603.00	25.00	1.72
	Average	62.59	218.02	407.36	71.09	3.09	356.31	14.41	1.16
	CV (%)	43.91	44.60	32.87	33.03	43.75	51.41	41.20	39.29
Total (n = 50)	Minimum	9.50	21.40	42.70	15.00	0.31	32.10	1.36	0.38
	Maximum	313.00	3810.00	4120.00	142.00	34.10	6150.00	296.00	6.43
	Average	87.92	613.08	867.61	68.26	6.81	1156.29	40.04	1.90
	CV (%)	78.14	132.98	98.67	34.00	105.11	107.50	136.33	73.37

**Table 2:** Total heavy metal(loid)s (mean values) in sediments from the Wushui river and other rivers in the Pearl River Basin.

River	Heavy Metal(loid) Concentration (mg/kg)								References
	Cu	Pb	Zn	Cr	Cd	As	Sb	Tl	
Wushui river	87.92	613.08	867.61	68.26	6.81	1156.29	40.04	1.90	This study
Diaojiang River, tributary of Pearl River	151.00	921.00	4314.00	64.2	4314.00	991.00	-	-	[45]
Mainstream of Pearl River	39.10	28.40	177.00	121.00	0.87	16.30	-	-	[45]
Hengshi River, tributary of Beijiang River	471.00	430.00	1601.00	-	6.96	104.00	-	0.26	[28]
Mainstream of Beijiang River	89.00	225.00	383.00	75.20	6.30	-	-	-	[25]
Background value	38.00	30.00	85.00	86.00	0.09	17.00	1.58	0.64	[44]

**Table 3:** Pearson correlation analysis among fractions of heavy metal(loid)s and physicochemical properties of sediments (n = 50).

Fraction	Sand	Silt	Clay	S	TN	TOC	pH	F1	F2	F3	F4
CuF1	−0.341	0.349 *	0.359 **	0.358 *	−0.244	−0.139	−0.205	1			
CuF2	−0.400 **	0.327 **	0.379 *	0.273	−0.227	0.022	0.017	0.780 **	1		
CuF3	−0.162	0.188	0.064	0.407 **	−0.290 *	−0.139	−0.034	0.575 **	0.748 **	1	
CuF4	−0.045	0.068	−0.023	0.593 **	−0.275	−0.032	−0.007	0.452 **	0.642 **	0.803 **	1
PbF1	0.043	−0.090	0.084	0.109	−0.280 *	−0.216	−0.048	1			
PbF2	−0.042	0.058	−0.007	0.712 **	−0.307 *	−0.112	−0.028	0.553 **	1		
PbF3	0.141	−0.154	−0.081	0.352 *	−0.287 *	−0.150	0.101	0.511 **	0.631 **	1	
PbF4	0.111	−0.087	−0.15	0.467 **	−0.246	−0.105	0.005	00.211	0.586 **	0.373 **	1
ZnF1	0.103	−0.107	−0.071	0.298 *	−0.329 *	−0.215	0.013	1			
ZnF2	0.047	−0.044	−0.048	0.317 *	−0.306 *	−0.180	0.101	0.907 **	1		
ZnF3	0.118	−0.112	−0.109	0.287 *	−0.366 **	−0.231	0.021	0.440 **	0.326 *	1	
ZnF4	0.062	−0.028	−0.137	0.814 **	−0.273	−0.050	−0.013	0.473 **	0.550 **	0.404 **	1
CdF1	0.014	−0.031	0.033	0.469 **	−0.377 **	−0.241	−0.109	1			
CdF2	0.063	−0.03	−0.137	0.617 **	−0.271	−0.079	0.127	0.731 **	1		
CdF3	0.127	−0.119	−0.124	0.218	−0.292 *	−0.172	0.034	0.284 *	0.208	1	
CdF4	0.064	−0.022	−0.157	0.811 **	−0.25	−0.047	−0.007	0.609 **	0.685 **	0.343 *	1
AsF1	0.005	−0.054	0.121	−0.035	−0.074	−0.118	−0.150	1			
AsF2	−0.091	0.059	0.154	−0.078	−0.159	−0.192	−0.170	0.576 **	1		
AsF3	0.117	−0.117	−0.096	0.079	−0.233	−0.200	−0.139	0.637 **	0.702 **	1	
AsF4	−0.031	0.058	−0.045	0.628 **	−0.319 *	−0.155	−0.050	00.277	0.468 **	0.474 **	1
SbF1	0.127	−0.169	0.003	−0.167	−0.231	−0.279 *	−0.213	1			
SbF2	0.010	−0.005	−0.022	0.283 *	−0.277	−0.164	−0.049	0.244	1		
SbF3	0.065	−0.083	−0.007	0.136	−0.309 *	−0.289	0.051	0.367 **	0.798 **	1	
SbF4	0.106	−0.084	−0.144	0.475 **	−0.259	−0.129	−0.003	0.158	0.881 **	0.679 **	1

* Correlation is significant at the 0.05 level. ** Correlation is significant at the 0.01 level.

**Table 4:** Summary of sediment guideline values and basic statistics of heavy metal(loid) in sediments.

		Pb	Zn	Cr	Cu	Cd	As	Sb	Tl
SQGs	TEC	35.8	121.0	43.4	31.6	0.99	9.79	NG	NG
	PEC	128.0	459.0	111.0	149.0	4.98	33.0	NG	NG
% of samples < TEC		2.0	6.0	14.0	16.0	6.0	0.0	-	-
% of samples between TEC–PEC		16.0	32.0	80.0	70.0	46.0	2.0	-	-
% of samples > PEC		82.0	62.0	6.0	14.0	48.0	98.0	-	-

TEC: a threshold effect concentration. PEC: a probable effect concentration. NG: no guideline.

**Table 5:** The statistical results of different assessment methods in the sediments.

Enrichment factor (EF)								
level	Cu	Zn	Pb	Cd	Cr	Sb	Tl	As
<1 (%)	14.0	0.0	6.0	76.0	0.0	0.0	0.0	2.0
1~2 (%)	30.0	12.0	10.0	24.0	0.0	0.0	14.0	40.0
2~5 (%)	40.0	12.0	22.0	0.0	4.0	10.0	6.0	46.0
>5 (%)	14.0	76.0	63.0	0.0	96.0	90.0	80.0	12.0
Geo-accumulation index (*I_geo_*)								
level	Cu	Pb	Zn	Cr	Cd	As	Sb	Tl
<0 (%)	30.0	2.0	10.0	96.0	0.0	0.0	2.0	22.0
0~1 (%)	22.0	14.0	10.0	4.0	2.0	4.0	12.0	50.0
1~2 (%)	38.0	6.0	30.0	0.0	2.0	0.0	6.0	18.0
2~3 (%)	10.0	28.0	26.0	0.0	12.0	18.0	24.0	10.0
3~4 (%)	0.0	16.0	18.0	0.0	14.0	6.0	18.0	0.0
4~5 (%)	0.0	26.0	6.0	0.0	24.0	22.0	26.0	0.0
>5 (%)	0.0	8.0	0.0	0.0	26.0	50.0	12.0	0.0
Risk assessment code (RAC)								
level	Cu	Zn	Pb	Cd	Cr	Sb	Tl	As
<1 (%)	6.0	0.0	6.0	0.0	2.0	12.0	18.0	46.0
1~10 (%)	78.0	14.0	78.0	0.0	98.0	86.0	82.0	48.0
11~30 (%)	12.0	60.0	16.0	14.0	0.0	2.0	0.0	6.0
31~50 (%)	2.0	22.0	0.0	48.0	0.0	0.0	0.0	0.0
>50 (%)	0.0	4.0	0.0	38.0	0.0	0.0	0.0	0.0

## Data Availability

Data will be made available on request.

## Supplementary Materials

The following supporting information can be downloaded at: https://www.mdpi.com/article/10.3390/toxics12020117/s1, Figure S1: The fractions distribution of heavy metal(loid)s in sediments of each site from Wushui River; Figure S2: BMI values for heavy metal(loid)s in the sediments of Wushui River; Table S1: Criteria of different assessment methods; Table S2: Sediment properties of Wushui River; Table S3: Pearson correlation analysis of heavy metal(loid)s in sediments (n = 50); Table S4: Principal component analysis for heavy metal(loid)s in sediments from Wushui River.

## References

[1] Xiao H., Shahab A., Xi B.D., Chang Q.X., You S.H., Li J.Y., Sun X.J., Huang H.W., Li X.K. (2021). Heavy metal pollution, ecological risk, spatial distribution, and source identification in sediments of the Lijiang River, China. Environ. Pollut..

[2] Resongles E., Casiot C., Freydier R., Dezileau L., Viers J., Elbaz-Poulichet F. (2014). Persisting impact of historical mining activity to metal (Pb, Zn, Cd, Tl, Hg) and metalloid (As, Sb) enrichment in sediments of the Gardon River, southern France. Sci. Total Environ..

[3] Debnath A., Singh P.K., Sharma J.C. (2021). Metallic contamination of global river sediments and latest developments for their remediation. J. Environ. Manag..

[4] Arisekar U., Shakila R.J., Shalini R., Jeyasekaran G., Sun S., Arumugam N., Almansour A., Perumal K. (2022). Potentially toxic elements contamination and its removal by aquatic weeds in the riverine system: A comparative approach. Environ. Res..

[5] Santos-Francés F., Martínez-Grāna A., Alonso R.P., García S.A. (2017). Geochemical background and baseline values determination and spatial distribution of heavy metal pollution in soils of the Andes Mountain range (Cajamarca-Huancavelica, Peru). Int. J. Environ. Res. Public Health.

[6] Salah E.A.M., Zaidan T.A., Al-Rawi A.S. (2012). Assessment of heavy metals pollution in the sediments of Euphrates River, Iraq. J. Water Resour. Prot..

[7] Xu F., Liu Z., Cao Y., Qiu L., Feng J., Xu F., Tian X. (2017). Assessment of heavy metal contamination in urban river sediments in the Jiaozhou Bay catchment, Qingdao, China. Catena.

[8] Javed T., Ahmad N., Mashiatullah A. (2018). Heavy metals contamination and ecological risk assessment in surface sediments of namal lake, Pakistan. Pol. J. Environ. Stud..

[9] Liu X.Y., Bai Z.K., Shi H.D., Zhou W., Liu X.C. (2019). Heavy metal pollution of soils from coal mines in China. Nat. Hazards.

[10] Long Z., Zhang W., Shi Z.L., Yu D.M., Chen Y., Liu C., Wang R. (2021). Effect of different industrial activities on soil heavy metal pollution, ecological risk, and health risk. Environ. Monit. Assess..

[11] Han Y., Gu X. (2023). Enrichment, contamination, ecological and health risks of toxic metals in agricultural soils of an industrial city, northwestern China. J. Trace Elem. Miner..

[12] Schäfer J., Counel A., Blanc G. (2022). Impact of metallurgy tailings in a major European fluvial-estuarine system: Trajectories and resilience over seven decades. Sci. Total Environ..

[13] Howard D.E., Evans R.D. (2010). Acid-volatile sulfide (AVS) in a seasonally anoxic mesotrophic lake: Seasonal and spatial changes in sediment AVS. Environ. Toxicol. Chem..

[14] Ganugapenta S., Nadimikeri J., Chinnapolla S.R.R.B., Ballari L., Madiga R., Nirmala K., Telia L.P. (2018). Assessment of heavy metal pollution from the sediment of Tupilipalem coast, southeast coast of India. Int. J. Sediment Res..

[15] Li Y., Lin Y., Wang L. (2018). Distribution of heavy metal(loid)sin seafloor sediments on the East China Sea inner shelf: Seasonal variations and typhoon impact. Mar. Pollut. Bull..

[16] Adimalla N., Chen J., Qian H. (2020). Spatial characteristics of heavy metal contamination and potential human health risk assessment of urban soils: A case study from an urban region of South India. Ecotoxicol. Environ. Saf..

[17] Zhang C., Shan B.Q., Zhao Y., Song Z.X., Tang W.Z. (2018). Spatial distribution, fraction, toxicity and risk assessment of surface sediments from the Baiyangdian Lake in northern China. Ecol. Indic..

[18] Aminiyan M.M., Aminiyan F.M., Mousavi R. (2016). Heavy metal pollution affected by human activities and different land-use in urban topsoil: A case study in Rafsanjan city, Kerman province, Iran. Eurasian J. Soil Sci..

[19] Zhao S., Feng C.H., Yang Y.R., Niu J.F., Shen Z.Y. (2012). Risk assessment of sedimentary metals in the Yangtze Estuary: New evidence of the relationships between two typical index methods. J. Hazard. Mater..

[20] Niu Y., Jiang X., Wang K., Xia J., Jiao W., Niu Y., Yu H. (2020). Meta analysis of heavy metal pollution and sources in surface sediments of Lake Taihu, China. Sci. Total Environ..

[21] Luo M.K., Yu H., Liu Q., Lan W., Ye Q.R., Niu Y., Niu Y. (2021). Effect of river-lake connectivity on heavy metal diffusion and source identification of heavy metals in the middle and lower reaches of the Yangtze River. J. Hazard. Mater..

[22] Vetrimurugan E., Jonathan M., Roy P.D., Shruti V., Ndwandwe O. (2016). Bioavailable metals in tourist beaches of Richards Bay, Kwazulu-Natal, South Africa. Mar. Pollut. Bull..

[23] Paul V., Sankar M.S., Vattikuti S., Dash P., Arslan Z. (2021). Pollution assessment and land use land cover influence on trace metal distribution in sediments from five aquatic systems in southern USA. Chemosphere.

[24] Gao B., Liang X., Zhou H. (2012). Lead isotopes as a tracer of Pb origin in the sediments from Beijiang River, South China. Water Sci. Technol..

[25] Li R., Tang C.Y., Li X., Jiang T., Shi Y., Cao Y. (2019). Reconstructing the historical pollution levels and ecological risks over the past sixty years in sediments of the Beijiang River, South China. Sci. Total Environ..

[26] Song M.W., Huang P., Li F., Zhang H., Xie K.Z., Wang X.H., He G.X. (2011). Water quality of a tributary of the Pearl River, the Beijiang, Southern China: Implications from multivariate statistical analyses. Environ. Monit. Assess..

[27] Liao J.B., Chen J., Ru X., Chen J.D., Wu H.Z., Wei C.H. (2017). Heavy metals in river sediments affected with multiple pollution sources, South China: Distribution, enrichment and source apportionment. J. Geochem. Explor..

[28] Luo C., Routh J., Dario M., Sarkar S., Wei L., Luo D.G., Liu Y. (2020). Distribution and mobilization of heavy metals at an acid mine drainage affected region in South China, a post-remediation study. Sci. Total Environ..

[29] Zhang J., Han L., Ji Y., Wei J., Cai G., Gao G., Wu J., Yao Z. (2018). Heavy metal investigation and risk assessment along the Le’An River from non-ferrous metal mining and smelting activities in Poyang, China. J. Environ. Biol..

[30] Liu B.X., Luo J., Jiang S., Wang Y., Li Y.C., Zhang X.S., Zhou S.Q. (2021). Geochemical fractionation, bioavailability, and potential risk of heavy metals in sediments of the largest influent river into Chaohu Lake, China. Environ. Pollut..

[31] Pueyo M., Mateu J., Rigol A., Vidal M., Lopez-Sanchez J.F., Rauret G. (2008). Use of the modified BCR three-step sequential extraction procedure for the study of trace element dynamics in contaminated soils. Environ. Pollut..

[32] MacDonald D.D., Ingersoll C.G., Berger T.A. (2000). Development and evaluation of consensus-based sediment quality guidelines for freshwater ecosystems. Arch. Environ. Contam. Toxicol..

[33] Smith S.L., Macdonald D.D., Keenleyside K.A., Ingeroll C.G., Field L.J. (1996). A preliminary evaluation of sediment quality assessment values for freshwater ecosystems. J. Great Lakes Res..

[34] Muller G. (1969). Index of geo-accumulation in sediments of the Rhine River. Geo J..

[35] Varol M. (2011). Assessment of heavy metal contamination in sediments of the Tigris River (Turkey) using pollution indices and multivariate statistical techniques. J. Hazard. Mater..

[36] Perin G., Craboledda L., Lucchese L., Cirillo R., Orio A.A. (1985). Heavy metal speciation in the sediments of northern Adriatic Sea. A new approach for environmental toxicity determination. Heavy Met. Environ..

[37] Sundaray S.K., Nayak B.B., Lin S., Bhatta D. (2011). Geochemical speciation and risk assessment of heavy metals in the river estuarine sediments-A case study: Mahanadi basin, India. J. Hazard. Mater..

[38] Ji Z.H., Zhang Y., Zhang H., Huang C.X., Pei Y.S. (2019). Fraction spatial distributions and ecological risk assessment of heavy metals in the sediments of Baiyangdian Lake. Ecotoxicol. Environ. Saf..

[39] Pavlovićs P., Markovićs M., Kostić O., Sakan S., Dordević D., Perović V., Pavlović D., Pavlović M., Cakmak D., Jarić S. (2019). Evaluation of potentially toxic element contamination in the riparian zone of the River Sava. Catena.

[40] Wu W.H., Qu S.Y., Nel W. (2020). The impact of natural weathering and mining on heavy metal accumulation in the karst areas of the Pearl River Basin, China. Sci. Total Environ..

[41] Huang B., Zhao Y.F., Shi X.Z., Yu D.S., Zhao Y.C., Sun W.X., Wang H.J. (2007). Source identification and spatial variability of nitrogen, phosphorus, and selected heavy metals in surface water and sediment in the riverine systems of a peri-urban interface. J. Environ. Sci. Health Part A.

[42] Li Z., Jiang W.G., Wang W.J., Chen Z., Ling Z.Y., Lv J.X. (2020). Ecological risk assessment of the wetlands in Beijing-Tianjin-Hebei urban agglomeration. Ecol. Indicat..

[43] Stevens H., Chase Z., Zawadzki A., Wong H., Proemse B.C. (2021). Reconstructing the History of Nutrient Loads and Sources in the Derwent Estuary, Tasmania, Australia, using Isotopic Fingerprinting Techniques. Estuar. Coast..

[44] Wang H., Wang J., Liu R., Yu W., Shen Z. (2015). Spatial variation, environmental risk and biological hazard assessment of heavy metals in surface sediments of the Yangtze River Estuary. Mar. Pollut. Bull..

[45] Wei B., Hou Q.Y., Tang Z.M. (2019). Estimation of Background Values and Contamination Characteristics of Heavy metals in Sediments of the Pearl River, China. Geoscience.

[46] Gusiatin Z.M., Kulikowska D. (2014). The usability of the IR, RAC and MRI indices of heavy metal distribution to assess the environmental quality of sewage sludge composts. Waste Manag..

[47] Feng C., Guo X., Yin S., Tian C.C., Li Y.Y., Shen Z.Y. (2017). Heavy metal partitioning of suspended particulate matter-water and sediment-water in the Yangtze Estuary. Chemosphere.

[48] Zhang G.L., Bai J.H., Xiao R., Zhao Q.Q., Cui B.S., Liu X.H. (2017). Heavy metal fractions and ecological risk assessment in sediments from urban, rural and reclamation-affected rivers of the Pearl River Estuary, China. Chemosphere.

[49] Pradit S., Shazili N.A.M., Pattaratumrong M.S., Chotikarn P., Towatana P. (2019). Chemical fractionation of trace elements in mangrove sediments from the Songkhla Lake, Thailand using BCR technique. Sci. Asia..

[50] Nemati K., Bakar N.K.A., Abas M.R., Sobhanzadeh E. (2011). Speciation of heavy metals by modified BCR sequential extraction procedure in different depths of sediments from Sungai Buloh, Selangor, Malaysia. J. Hazard. Mater..

[51] Parizanganeh A. Grain size effect on trace metals in contaminated sediments along the Iranian coast of the Caspian Sea. Proceedings of the Taal 2007 12th World Lake Conference.

[52] Mahdi A.M., Doumenq P., Awaleh M.O., Syakti A.D., Asia L., Chiron S. (2017). Levels and sources of heavy metals and PAHs in sediment of Djibouti-city (Republic of Djibouti). Mar. Pollut. Bull..

[53] Ren Y.Y., Cao X.X., Wu P., Li L.W. (2023). Experimental insights into the formation of secondary minerals in acid mine drainage-polluted karst rivers and their effects on element migration. Sci. Total Environ..

[54] Fang X.H., Peng B., Wang X., Song Z.L., Zhou D.X., Wang Q., Qin Z.L., Tan C.Y. (2019). Distribution, contamination and source identification of heavy metals in bed sediments from the lower reaches of the Xiangjiang River in Hunan province, China. Sci. Total Environ..

[55] Liang G., Zhang B., Lin M., Wu S., Hou H., Zhang J., Qian G., Huang X., Zhou J. (2017). Evaluation of heavy metal mobilization in creek sediment: Influence of RAC values and ambient environmental factors. Sci. Total Environ..

[56] Zhuang Q., Li G., Liu Z. (2018). Distribution, source and pollution level of heavy metals in river sediments from South China. Catena.

[57] Wang X., He M., Xi J., Lu X. (2011). Antimony distribution and mobility in rivers around the world’s largest antimony mine of Xikuangshan, Hunan Province, China. Microchem. J..

