# Supplementary material for: Distribution, Ecological Risk, and Source Identification of Heavy Metal(loid)s in Sediments of a Headwater of Beijiang River Affected by Mining in Southern China
Source: Toxics. 2024 Jan 30;12(2):117. doi: 10.3390/toxics12020117 (PMC10892579; doi:10.3390/toxics12020117)
Supplement: Supplementary file 1 [file toxics-12-00117-s001.zip › toxics-2822090-supplementary.pdf]

Supplementary Materials

Table S1. Criteria of different assessment methods.

**Geo-accumulation index (I_{geo})**	
**I_{geo}**	**Pollution level**
< 0	Unpolluted
0–1	Unpolluted to moderately polluted
1–2	Moderately polluted
2–3	Moderately to strongly polluted
3–4	Strongly polluted
4–5	Strongly to very strongly polluted
≥ 5	Very strongly polluted
**Enrichment factor (EF)**	
**EF**	**Pollution level**
<1	deficient enrichment
1≤EF<2	minimal enrichment
2≤EF<5	moderate enrichment
EF≥5	significantly enriched
**Risk assessment code (RAC)**	
**RAC**	**Sediment risk**
<1	No risk
1-10	Low risk
11-30	Medium risk
31-50	High risk
≥50	Very high risk

Table S2. Sediment properties of Wushui River.

River sections	Item	pH	TOC(%)	TN(mg kg^{-1})	S(%)	Sand (%)	Silt (%)	Clay (%)
Zone1(n=4)	Maximum	8.30	2.17	1020.00	0.10	87.00	22.80	21.10
	Minimum	5.50	0.31	394.00	0.02	56.10	7.00	5.90
	Average	7.48	0.91	661.00	0.06	77.70	11.48	10.83
	CV（%）	17.69	93.96	41.64	54.50	18.85	66.05	66.06
Zone2(n=15)	Maximum	8.90	2.07	1520.00	0.94	99.80	67.90	25.30
	Minimum	5.20	0.24	117.00	0.05	6.80	0.20	0.00
	Average	7.69	0.88	544.40	0.39	70.76	20.53	8.71
	CV（%）	10.17	72.48	89.25	66.86	36.84	96.00	83.49
zone3(n=6)	Maximum	8.00	2.43	743.00	4.50	87.70	39.10	15.90
	Minimum	7.40	0.67	161.00	0.26	45.00	8.30	3.30
	Average	7.72	1.45	379.67	1.54	66.57	24.90	8.53
	CV（%）	2.65	49.00	55.54	115.84	26.15	49.27	63.97
Zone4(n=9)	Maximum	10.60	2.55	1700.00	2.76	85.80	36.60	29.30
	Minimum	3.10	1.79	152.00	0.03	34.10	9.20	2.20
	Average	7.43	0.37	976.22	0.49	70.27	20.97	8.77
	CV（%）	27.79	46.00	60.66	181.57	22.40	45.47	93.06
Zone5(n=3)	Maximum	8.10	1.94	483.00	1.79	84.40	18.20	7.40
	Minimum	7.70	0.66	202.00	0.04	74.40	10.70	4.90
	Average	7.93	1.19	360.33	0.73	78.70	15.17	6.13
	CV（%）	2.62	55.82	39.92	128.40	6.54	26.04	20.39
Zone6(n=2)	Maximum	8.10	1.31	1100.00	0.21	87.50	29.30	11.40
	Minimum	7.80	0.95	956.00	0.04	59.30	5.70	6.80
	Average	7.95	1.13	1028.00	0.12	73.40	17.50	9.10
	CV（%）	2.67	22.38	9.90	100.03	27.17	95.36	35.74
Zone7(n=11)	Maximum	8.60	2.87	3860.00	0.28	97.00	62.50	18.90
	Minimum	5.90	0.92	436.00	0.04	18.60	1.90	1.10
	Average	7.90	1.73	1536.36	0.16	69.95	22.12	7.93
	CV（%）	0.09	0.43	0.71	0.55	0.39	0.99	0.74
Total(n=50)	Maximum	10.60	2.87	3860.00	4.50	99.80	67.90	29.30
	Minimum	3.10	0.24	117.00	0.02	6.80	0.20	0.00
	Average	7.70	1.33	838.22	0.48	71.13	20.32	8.56
	CV（%）	13.77	59.00	89.17	174.72	29.86	79.41	74.19

Table S3. Pearson correlation analysis of heavy metal(loid)s in sediments (n=50).

	Cu	Pb	Zn	Cr	Cd	As	Sb	Tl
Cu	1							
Pb	.626**	1						
Zn	.561**	.766**	1					
Cr	-0.274	-0.268	-.397**	1				
Cd	.605**	.847**	.962**	-.310*	1			
As	.839**	.755**	.676**	-0.237	.727**	1		
Sb	.676**	.851**	.706**	-0.209	.728**	.779**	1	
Tl	0.277	0.225	0.24	-0.239	0.179	.285*	0.111	1

*Correlation is significant at the 0.05 level. ** Correlation is significant at the 0.01 level.

Table S4. Principal component analysis for heavy metal(loid)s in sediments from Wushui River.

Component	Initial eigenvalue			Component matrix		
	Total	Variance %	Cumulative %	Total	Variance %	Cumulative %
1	4.917	61.462	61.462	4.917	61.462	61.462
2	1.075	13.440	74.902	1.075	13.440	74.902
3	.824	10.306	85.208	.824	10.306	85.208
4	.586	7.328	92.536			
5	.316	3.945	96.481			
6	.137	1.711	98.192			
7	.131	1.641	99.833			
8	.013	.167	100.000			

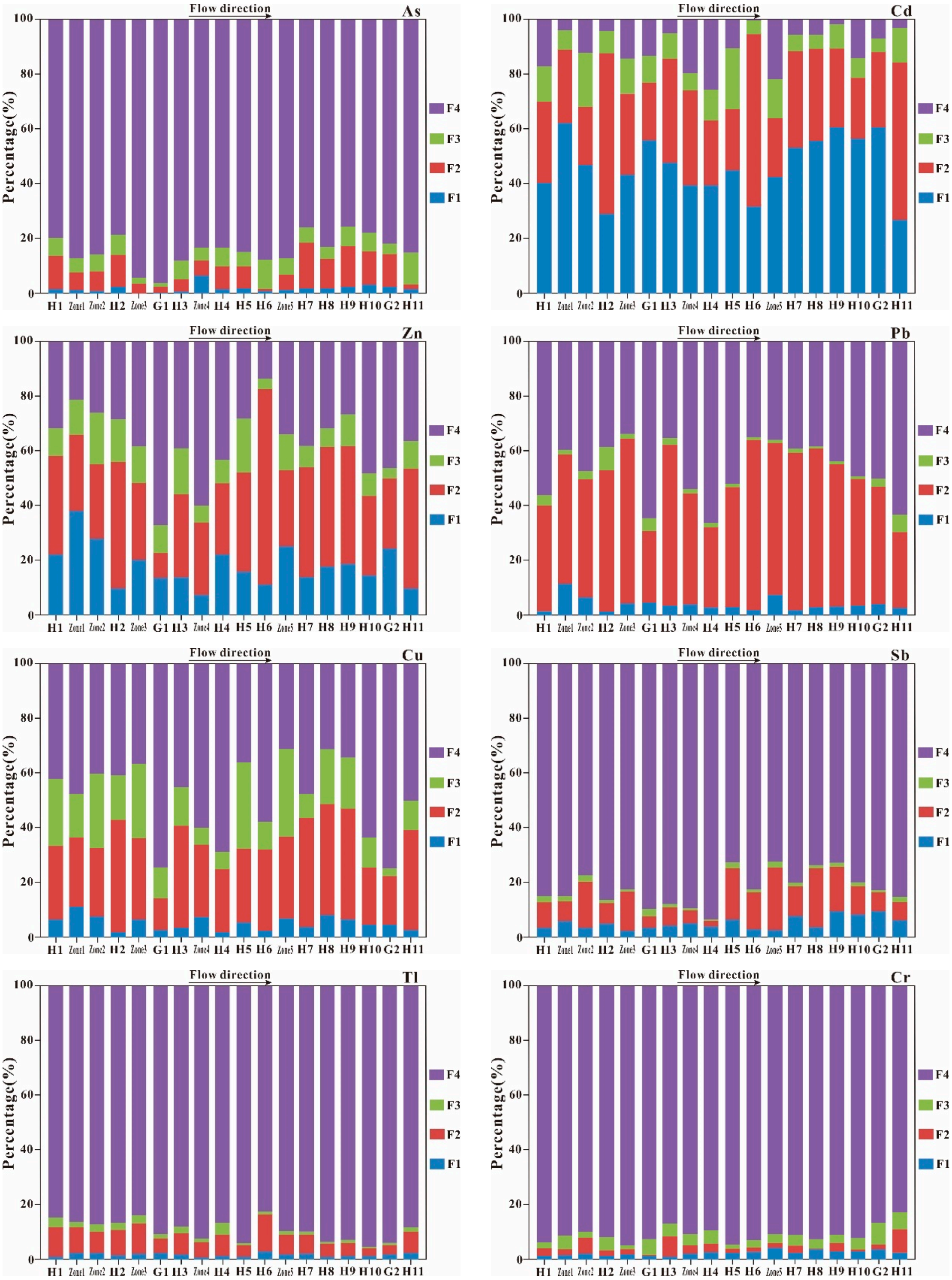

Figure S1. The fractions distribution of heavy metal(loid)s in sediments of each site from Wushui River.

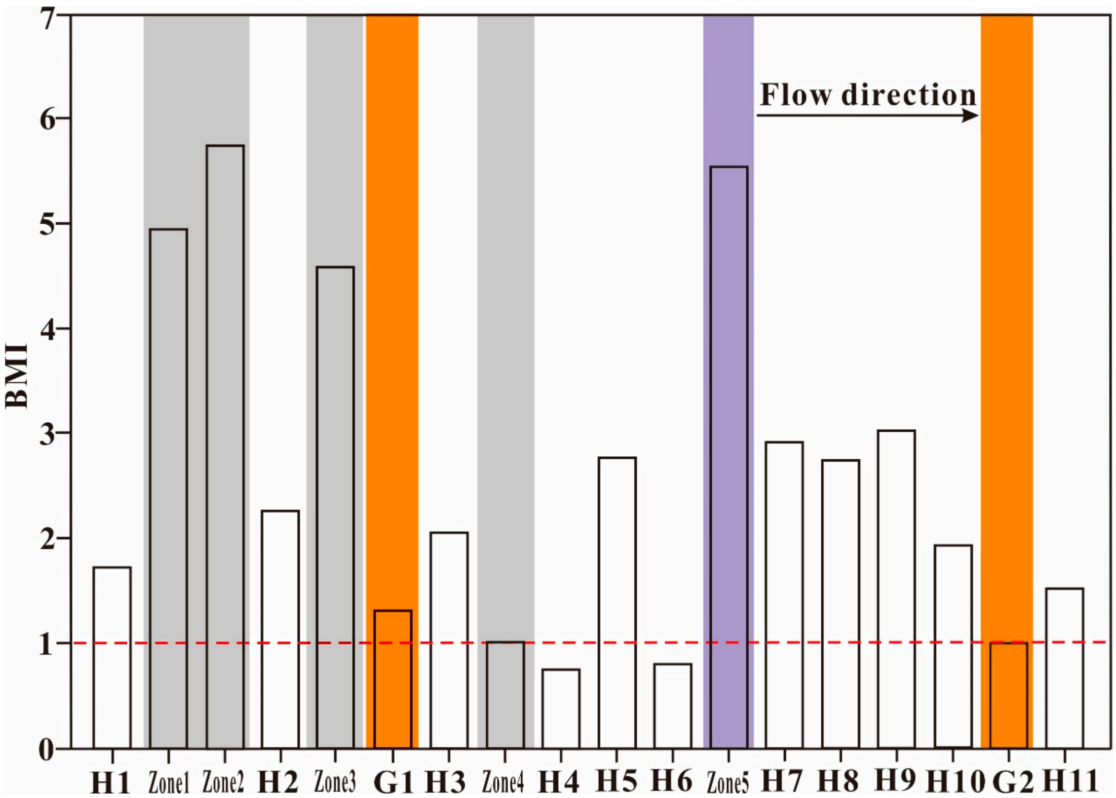

Figure S2. BMI values for heavy metal(loid)s in the sediments of Wushui River.
